# The complete mitochondrial genome of *Metasepia tullbergi* (Cephalopoda: Sepiidae)

**DOI:** 10.1080/23802359.2021.1902873

**Published:** 2021-03-26

**Authors:** Hung-Tai Lee, Cheng-Hsin Liao, Chang-Wen Huang, Chia-Huan Ma, Te-Hua Hsu

**Affiliations:** a Department of Environmental Biology and Fisheries Science, National Taiwan Ocean University, Keelung, ROC; b Department of Aquaculture, National Taiwan Ocean University, Keelung, ROC; c Center of Excellence for the Oceans, National Taiwan Ocean University, Keelung, ROC

**Keywords:** Cuttlefish, phylogenetic analysis, mitogenome, next-generation sequencing

## Abstract

The first complete mitochondrial genome of *Metasepia tullbergi* has been characterized in this study. The circular mitogenome is 16182 bp in length and comprises 13 protein-coding genes (PCGs), 22 transfer *RNA* genes, and two ribosomal *RNA* genes. The organization of these genes is highly consistent with that of other Sepiidae. The overall base composition of mitogenome is 39.20% A, 36.07% T, 8.98% G, and 15.75% C, with 75.27% AT. Phylogenetic analysis further suggests that *M. tullbergi* is placed within the Sepiidae and is closely related to *Sepia latimanus* and *S. apama*.

The paintpot cuttlefish (*Metasepia tullbergi*) is a small cuttlefish belonging to the Sepiidae family. It is a neritic demersal species that inhabits the sandy and muddy continental shelf with a water depth of 20–100 m. *M. tullbergi* commonly appears in the Indo-Pacific region, including Japan, Korea, China, Hong Kong, Taiwan, the Philippines, and the Gulf of Thailand (Reid et al. 2005). As a member of the *Metasepia* genus, *M. tullbergi* has a small and thick, diamond-shaped cuttlebone and exhibits a unique body coloration (Thomas and MacDonald 2016). In this study, we aim to report the first complete mitochondrial genome of *M. tullbergi* and further analyze its phylogenetic relationship within the family Sepiidae.

The specimen of *M. tullbergi* was collected from the coastal water off northeastern Taiwan (121.9°E, 25.1°N) in May of 2020 and stored at National Taiwan Ocean University with a specimen number (NTOU-MT-01-2020). The total genomic DNA was prepared and then followed by the pair-end sequencing (2 × 150 bp) with Novaseq (Illumina, San Diego, CA). The *de novo* assembly of the complete mitochondrial genome of *M. tullbergi* was performed using Geneious Prime 2020.2 (Kearse et al. 2012). The identification and annotation of protein-coding genes (PCGs) were conducted using ORFfinder (https://www.ncbi.nlm.nih.gov/orfnder). Additionally, the transfer RNA (*tRNA*) and ribosomal RNA (*rRNA*) genes were identified and annotated using MITOS Web Server (Bernt et al. 2013).

The complete mitochondrial genome of *M. tullbergi* was a closed-circular molecule with 16,182 bp in length (GenBank accession number: MT974497). It contains 13 PCGs, 22 transfer *RNA* genes (tRNAs), two ribosomal *RNA* genes (12S rRNA and 16S rRNA). The overall base composition of mitochondrial genome is biased toward A + T content at 75.27% (A = 39.20%, T = 36.07%, G = 8.98%, and C = 15.75%). The length of 13 PGCs ranges from 156 to 1749 bp. All PCGs initiate with ATG. 9 PCGs terminate with TAA while 4 PCGs (ATP6, NAD1, CYTB, and NAD6) terminate with TAG. The length of the 22 *tRNA* genes ranges from 60 to 73 bp. All *tRNA* genes possess a typical cloverleaf-shaped secondary structure. The 16S rRNA with a length of 1061 bp is located between trnL and trnV. The 12S rRNA with a length of 891 bp is located between trnV and trnC.

The phylogenetic position of *M. tullbergi* was further examined based on a maximum-likelihood phylogenetic tree constructed by 13 PCGs in the complete mitochondrial genomes of *M. tullbergi* and other closely related species using MEGA X (Kumar et al. 2018). The result indicated that *M. tullbergi* clustered within the Sepiidae and was closely related to *Sepia latimanus* and *S. apama* (Figure 1).

**Figure 1.**
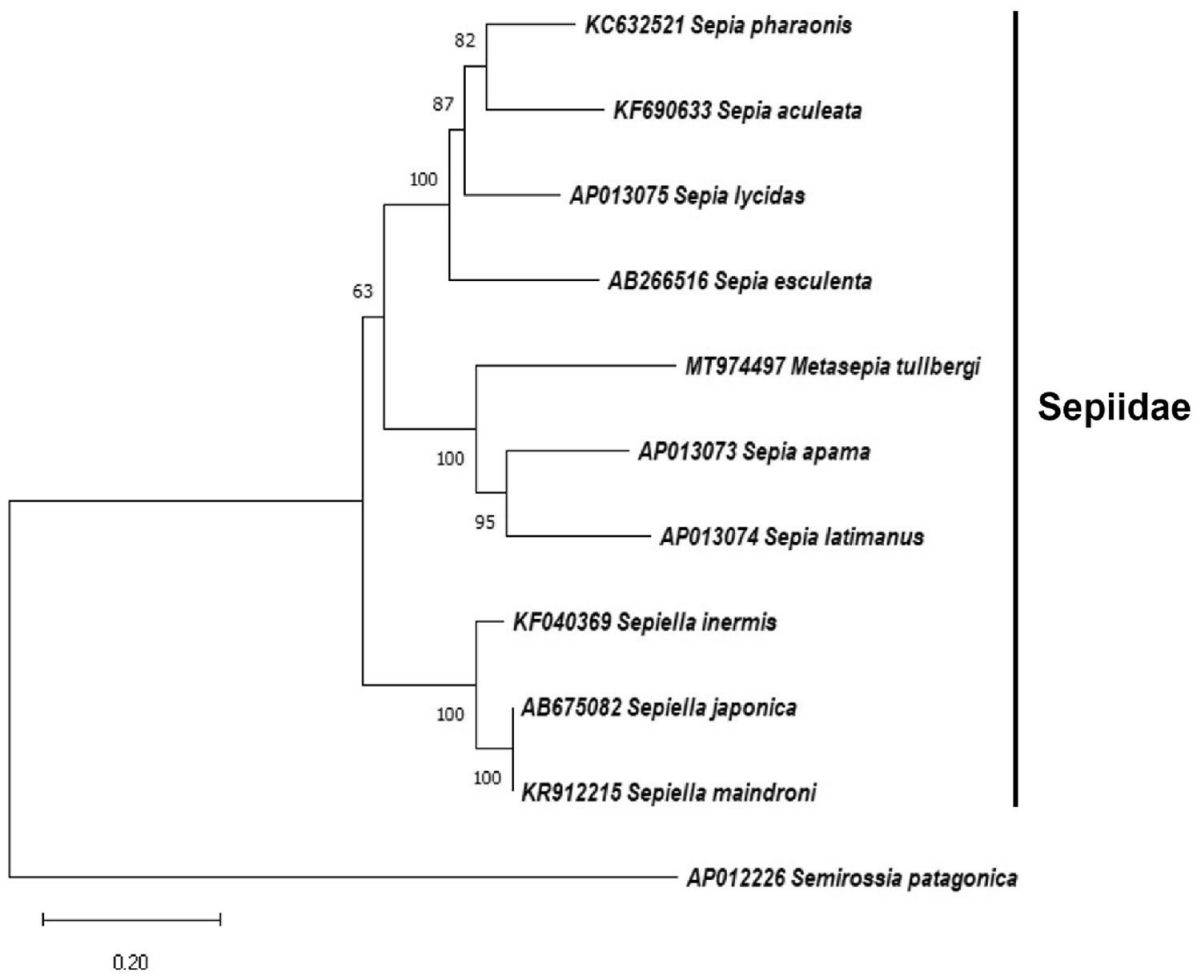
Maximum-likelihood phylogenetic tree constructed by 13 PCGs in the mitochondrial genome of *Metasepia tullbergi* and the other nine Sepiidae species. *Semirossia patagonica* is used as the outgroup. Numbers beside each node represent percentages of 1000 bootstrap values.

## Data Availability

The data that support the findings of this study are publicly available in GenBank of NCBI at https://www.ncbi.nlm.nih.gov, Accession number: MT974497. The associated BioProject, SRA, and Bio-Sample numbers are PRJNA679401, SRX9530327, SAMN16832771.
